# Quantitative proteomics and terminomics to elucidate the role of ubiquitination and proteolysis in adaptive immunity

**DOI:** 10.1098/rsta.2015.0372

**Published:** 2016-10-28

**Authors:** Theo Klein, Rosa I. Viner, Christopher M. Overall

**Affiliations:** 1 Centre for Blood Research, University of British Columbia, Vancouver, BC Canada V6T 1Z3; 2 Department of Oral Biological and Medical Sciences, Faculty of Dentistry, University of British Columbia, Vancouver, BC Canada V6T 1Z3; 3 Department of Biochemistry and Molecular Biology, University of British Columbia, Vancouver, BC Canada V6T 1Z3; 4 Thermo Fisher Scientific, San Jose, CA 95134, USA

**Keywords:** ubiquitin, linear ubiquitin, LUBAC, proteases, MALT1, degradomics

## Abstract

Adaptive immunity is the specialized defence mechanism in vertebrates that evolved to eliminate pathogens. Specialized lymphocytes recognize specific protein epitopes through antigen receptors to mount potent immune responses, many of which are initiated by nuclear factor-kappa B activation and gene transcription. Most, if not all, pathways in adaptive immunity are further regulated by post-translational modification (PTM) of signalling proteins, e.g. phosphorylation, citrullination, ubiquitination and proteolytic processing. The importance of PTMs is reflected by genetic or acquired defects in these pathways that lead to a dysfunctional immune response. Here we discuss the state of the art in targeted proteomics and systems biology approaches to dissect the PTM landscape specifically regarding ubiquitination and proteolysis in B- and T-cell activation. Recent advances have occurred in methods for specific enrichment and targeted quantitation. Together with improved instrument sensitivity, these advances enable the accurate analysis of often rare PTM events that are opaque to conventional proteomics approaches, now rendering in-depth analysis and pathway dissection possible. We discuss published approaches, including as a case study the profiling of the N-terminome of lymphocytes of a rare patient with a genetic defect in the paracaspase protease MALT1, a key regulator protease in antigen-driven signalling, which was manifested by elevated linear ubiquitination.

This article is part of the themed issue ‘Quantitative mass spectrometry’.

## 1. Introduction

Vertebrates possess a powerful arsenal of defence mechanisms against pathogens, consisting of two seemingly separate, but finely intertwined, immune systems. As a first line of defence, the relatively unspecific innate immune system detects structural components of microorganisms termed pathogen-associated molecular patterns (PAMPs). Innate immune cells are equipped with a range of pattern recognition receptors (PRRs) such as the Toll-like receptor family that activates immune signalling cascades within the cells upon ligation of the PAMP receptor, leading to an inflammatory response and elimination of the invading pathogen (reviewed in [1]). The second system consists of lymphocyte-mediated adaptive immunity where receptors on B and T cells recognize specific protein antigens, which leads to mounting a powerful and highly selective immune response. During early development of the immune system, a process of genetic recombination in progenitor cells generates a large ‘library’ of lymphocytes with membrane-bound immunoglobulin or B-cell receptor (BCR), or T-cell receptors (TCRs) with a unique specificity. Ligation of a lymphocyte receptor with its corresponding antigen sets in motion a complex and highly regulated intracellular signalling cascade that leads to activation of the transcription factor nuclear factor-kappa B (NF-κB) and subsequent production of pro-inflammatory mediators, and factors promoting proliferation and maturation of the activated lymphocytes. A sequence of several specific post-translational modifications (PTMs), such as phosphorylation, ubiquitination events and specific protease cleavages termed proteolytic processing and non-specific proteolytic degradation by the proteasome, are required to mount and maintain a full response. In this review, we discuss targeted quantitative proteomics approaches to investigate these processes.

## 2. Ubiquitination in adaptive immunity

**Figure 1.**
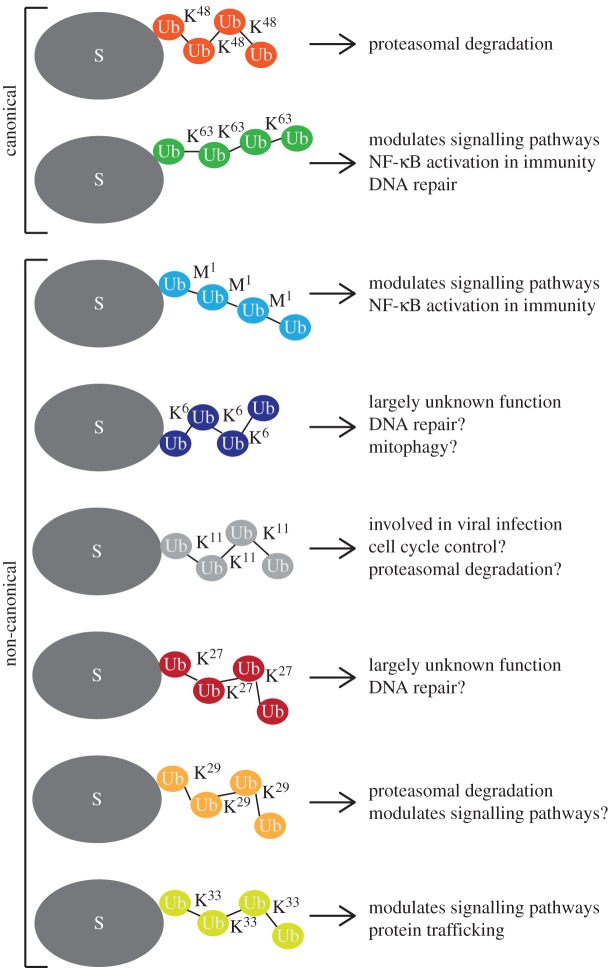
Scheme displaying different homotypic polyubiquitin chains conjugated to protein substrates *in vivo* and biological outcome of ubiquitination. Ub, ubiquitin monomer; S, substrate. (Online version in colour.)

Ubiquitination is a common and reversible PTM where the 8.5 kDa ubiquitin protein is ligated to a target lysine residue in a substrate protein. This process is orchestrated by a coordinated three-step reaction of ubiquitin-activating (E1), ubiquitin-conjugating (E2) and ubiquitin-ligating (E3) enzymes [2]. The human proteome contains at least 600 unique E3 ligases that mainly determine the specificity of the ubiquitination cascade, many of which have no known targets. Ligation of an initial ubiquitin group to a substrate protein can lead to formation of a polyubiquitin chain through formation of isopeptide bonds between the C-terminal glycine in an additional ubiquitin molecule and either to the ϵ-amino group in one of the seven internal lysine residues (K6, K11, K27, K29, K33, K48, K63) or to the N-terminal methionine α-amino group (M1) of the bound ubiquitin, forming a polymer of linear ubiquitin. The exact conformation of the polyubiquitin chain determines the fate of the substrate: K48 chains generally target the substrate for degradation by the proteasome; K63 and M1 have specific roles in protein : protein interaction in signal transduction (figure 1, and reviewed in [3,4]). Polyubiquitin chains can be removed from substrates by a family of 104 cysteine- and metalloproteases, named deubiquitinases (DUBs), that have specificities for different ubiquitin chain structures. As with E3 ligases, their substrates remain unclear, although large-scale interactomics studies have shed some light on this [5].

Regulated ubiquitination of key substrates is critical in most signalling pathways in both innate and adaptive immunity (reviewed in [6]), including canonical NF-κB activation upon antigen receptor ligation. E3 ligases in the Cbl family are negative regulators by conferring K48 chains to substrates or blocking interaction sites for activating kinases in the antigen receptor signalosome [7,8]. After a series of activating phosphorylations, the antigen signal reaches a control hub in the pathway; a trimeric complex consisting of caspase recruitment domain-containing protein 11 (CARD11), B-cell lymphoma protein 10 (BCL10) and the unique paracaspase mucosa-associated lymphoid tissue lymphoma translocation protein 1 (MALT1), known as the CBM complex. Monoubiquitination of MALT1 is required for activity [9], and once formed the CBM attracts other E3 ligases to the signalosome, including tumour necrosis factor (TNF)-receptor associated factor-6 (TRAF6) and Mindbomb-2 (MIB2) that subsequently form K63 chains on NF-κB essential modulator (NEMO), which activates the IκB kinase (IKKα/β) complex that phosphorylates NF-κB/p65, leading to K48 ubiquitination and proteasomal degradation of the inhibitor IκBα. Phosphorylated NF-κB translocates to the nucleus to activate transcription (reviewed in [10]). DUBs that target these substrates are negative regulators: A20 hydrolyses K63 polyubiquitin chains, including on MALT1, to dampen the NF-κB response. CYLD removes K63 chains from NEMO and other targets, and is one of only two DUBs known to cleave M1 linear ubiquitin chains, besides OTULIN that exclusively cleaves linear ubiquitin [11,12].

## 3. Applications of proteomics techniques to elucidate the role of ubiquitin in lymphocyte antigen signalling

Proteomic profiling of interactors with known signalling proteins has identified unexpected ubiquitin-related proteins, painting a picture of a far more complicated and intertwined regulatory mechanism than often assumed. Interactors of E3 ligase Cbl and its known interactor CIN85 associate with the phosphatase SHIP-1 upon BCR cross-linking, epitomizing cross-talk between phosphorylation and ubiquitination [13]. Mass spectrometry (MS) analysis of proteins co-immunoprecipitated with a BCL10-GyrB fusion protein mimicking activation and dimerization upon treatment with coumermycin in various T-cell lines revealed that the ubiquitin E3 ligase MIB2 associated with BCL10 in activated T cells, and activated the IKK complex by direct or indirect ubiquitination of NEMO. MIB2 knockout cells showed reduced NF-κB activation upon TCR, but not TNFα stimulation. Thus, unexpectedly, not only TRAF6 but also MIB2 is an E3 ligase that mediates signal transduction in TCR signalling [14]. Interactomics of casein kinase-1α (CK1α), an upstream kinase in TCR signalling, revealed that the E3 ligase HOIP associated with CK1α and the CBM complex in a large signalosome upon TCR activation [15]. HOIP is a component of linear ubiquitin chain assembly complex (LUBAC) with HOIL-1 and SHARPIN that forms linear (M1) ubiquitin chains important in immunity (reviewed in [16]). LUBAC has been studied in TNFα, CD40 and interleukin-1β signalling in lymphocytes but has unclear roles in antigen signalling.

The crucial role of ubiquitination in immunity, combined with knowledge gaps, makes this system an interesting target for unbiased quantitative proteomics profiling of antigen receptor signalling. Ubiquitin proteomics has been hard to achieve. The high proteolytic activity of DUBs makes preserving the ubiquitination status after cell disruption challenging, and due to the low relative abundance of specific ubiquitination events compared with the total protein pool, a selective enrichment of ubiquitinated proteins is desired prior to MS analysis. Ubiquitin-modified proteins yield specific peptides upon tryptic digestion of the proteome; the modified lysine residue will retain a diglycyl isopeptide remnant that can be identified by MS. Two potential pitfalls are present, however; first, the diglycyl remnant is not unique to ubiquitinated proteins but also results from proteins modified with the less abundant ubiquitin-like modifiers interferon-stimulated gene-15 (ISG15) and NEDD8, so further validation is required. Secondly, the mass shift due to the ubiquitin remnant is identical to an artefact modification of lysine occurring during carbamidomethylation to block thiol groups on cysteine residues, which therefore requires careful sample handling, temperature control and data analysis [17].

**Figure 2.**
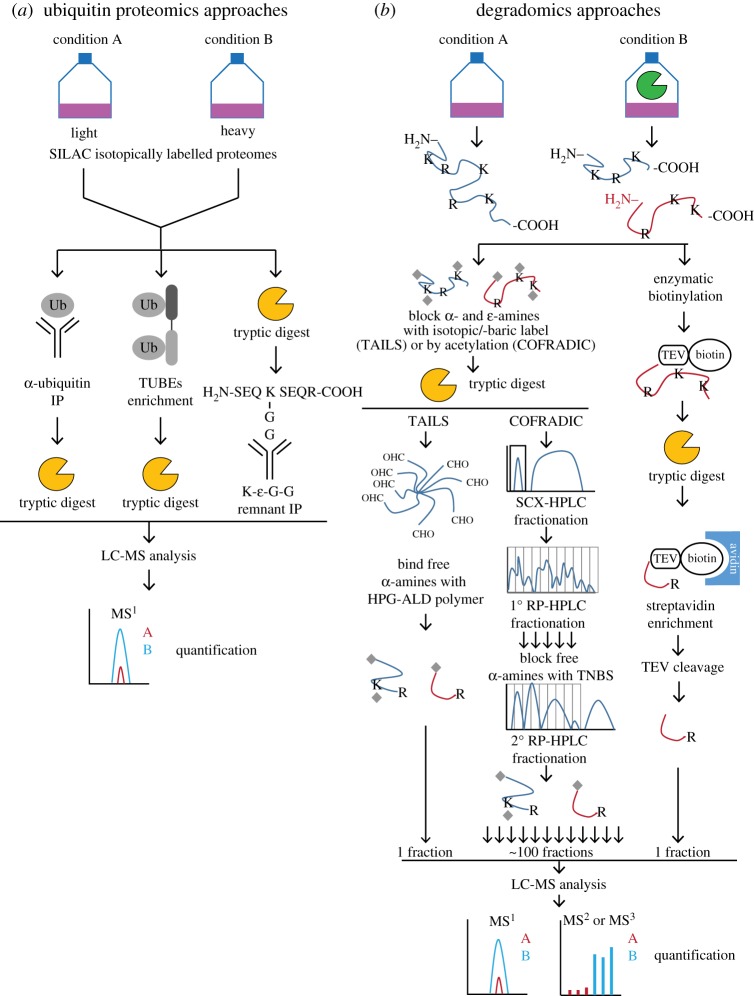
Schematic overview of quantitative proteomics methods discussed in this paper. (*a*) Workflow of enrichment methods used in ubiquitin-profiling proteomics based on anti-ubiquitin (Ub) immunoprecipitation (left), ubiquitin pull down with selective ubiquitin-binding domain reagents (TUBEs, middle) and tryptic ubiquitin remnant peptide antibody immunoprecipitation (right). (*b*) Workflow of methods for enriching N-terminal peptides by chemical labelling of primary amines followed by removing non-labelled peptides via binding to an aldehyde-functionalized polymer (TAILS, left), chemical labelling of primary amines and sequential chromatographic fractionation to enrich for N-termini (COFRADIC), and enzymatic labelling of N-terminal α-amines and subsequent pull down of labelled peptides (right). (Online version in colour.)

Affinity enrichments of the pool of ubiquitinated proteome (ubiquitome) followed by MS analysis have been applied with varying degrees of success (figure 2*a* and reviewed in [18]). Combining affinity pull down and MS conformation of the ubiquitination site is capable of generating datasets comparable in size to those in phosphoproteomics. Initial attempts using (poly)ubiquitin-specific antibodies for immunoprecipitation identified hundreds of ubiquitinated proteins [19], but as with all protein-level pull downs suffered from cross-reactivity and non-specific interactions. A commonly used strategy to improve selectivity is transfection with an affinity-tagged (e.g. FLAG or 6xHis) ubiquitin construct to enable more efficient enrichment [20]. One could argue that by perturbing the system, not all observed substrates may be physiological, and the effect of an affinity tag on formation of polyubiquitin chains is unclear. Improved affinity and selectivity can also be achieved by using natural ubiquitin-interacting domains called ubiquitin-associated domains (UBAs) that can selectively bind different ubiquitin topologies [21]. Combining several domains in a single affinity ligand such as tandem ubiquitin-binding entities (TUBEs [22]) or recently tandem hybrid ubiquitin-binding domains (ThUBDs [23]) can provide effective enrichment, allowing in-depth profiling of one or more ubiquitin chain types.

Several approaches have used DUBs to probe the ubiquitinated proteome. By using a set of DUBs with assumed chain topology specificity, ubiquitin chain restriction (UbiCRest) provides the opportunity to investigate the different homo- and heterotypic polyubiquitin chains that are conjugated to substrates [24]. The Gevaert lab has linked removal of all conjugated ubiquitin in a sample by the promiscuous enzyme USP2, followed by chemical modification of the formerly modified lysine residue, to a series of chromatographic fractionations into 60 final fractions to enrich for the modified peptides, allowing identification of more than 7500 ubiquitination sites from 4 mg of proteome from Jurkat T cells [25].

The largest breakthrough in ubiquitin proteomics was achieved by the generation of a specific antibody against the diglycyl-lysine group that results from tryptic digestion of a ubiquitinated protein to allow enrichment of remnant containing peptides [26–28]. By using differential isotopic labelling, e.g. by SILAC (stable isotope labelling with amino acids in cell culture), this method is capable of quantifying more than 10 000 ubiquitination sites in a biological sample, allowing powerful profiling and substrate discovery for even very specific E3 ligases or DUBs [29–31]. In a large-scale SILAC quantitative proteomics study investigating the effect of a spleen tyrosine kinase (Syk) inhibitor on phosphorylation events downstream of BCR ligation, the Tao lab found an interesting cross-talk between phosphorylation and ubiquitination, where Syk-dependent phosphorylation events in ubiquitin ligases and DUBs were required for upregulated total ubiquitination. Diglycine remnant antibody proteomics identified close to 1000 ubiquitination sites to be up- or downregulated upon Syk inhibition, indicating the scale and importance of ubiquitination as a regulator in antigen signalling [32]. In a recent study, the Choudhary lab combined interactomics, phosphoproteomics and ubiquitin remnant pull-down proteomics in a systems biology approach to elucidate the events following BCR engagement in murine B-cell lymphoma cells [33]. Pull down of the BCR signalosome with α-IgG revealed at least 154 interactors upon receptor engagement, many of which were not known to play a role in antigen signalling. Analysis of the ubiquitome in the same cells revealed 250 ubiquitination sites to be upregulated even within 5 min of BCR ligation; many of the substrates were known to be functionally relevant in the BCR signalling pathway, including the negative regulator A20. Performing TUBEs enrichment of the ubiquitome on the protein level, followed by targeted LC-MS/MS, the authors identified each polyubiquitin linkage. Interestingly, upon pull down of the ubiquitinated pool, mainly linear ubiquitin was upregulated after BCR activation including on BCL10 in the CBM.

This was an interesting finding, since linear ubiquitination has been reported in many other immune signalling pathways, but was never assumed to be relevant in antigen receptor stimulation. Linear ubiquitination of substrates like NEMO seems to play an important role in scaffolding of the receptor signalosome, and the identification of BCL10 as an additional target in this pathway opens up the possibility of a much larger role for LUBAC in lymphocytes than previously thought. Many of the E3 ligases (such as the TRAF family) share a common role in different pathways, even though stimulation may result in different cellular outcomes. Analysis of potential differential substrate specificity depending on the receptor that is triggered could provide interesting biological insights.

## 4. Proteolysis in adaptive immunity

Proteolysis is an irreversible PTM [34] and is arguably the commonest PTM since every protein molecule will be targeted during its cellular life. Proteolysis is often associated with mere degradation or protein turnover, but targeted, specific proteolytic events known as processing are key regulators of the biological activity of many proteins such as cytokines, chemokines [35], growth factors and proteases themselves [36]. Dysregulation of proteolysis is a key in many pathologies, and proteases are considered attractive, although sometimes challenging, targets for pharmaceutical intervention [37,38].

Proteases play a pivotal role in the regulation of the adaptive immune response, both in activation upon challenge and in maintenance of a proper immune repertoire. Major histocompatibility complex (MHC) presentation of antigens by antigen-presenting cells (APCs) depends on regulated proteolysis of intracellular or internalized proteins into MHC-presentable peptides by the immuno-proteasome (for MHC-I) or proteolysis by legumain and cathepsins (in case of MHC-II). Legumain also activates the MHC-II protein complex inside the MHC compartment, to allow loading of the newly generated peptide (reviewed in [39]). Proteases are effector proteins in CD8^+^ cytotoxic T cells, and caspases regulate both maturation of lymphocytes and regulated removal of the expanded specific lymphocyte population by apoptosis after the immune challenge has resolved (reviewed in [40]). MALT1 is a critical regulator of lymphocyte activation; through formation of the CBM complex, it allows transduction of the signal from activated antigen receptors in B and T cells, and modulates the signal through cleavage of several inhibitory factors such as A20 and CYLD (reviewed in [41]). Finally, MALT1 was recently shown by N-terminomic and proteomic analyses of B cells to regulate the dampening of the antigen signal through cleavage of HOIL1, deactivating and disassembling LUBAC, and abrogating signal-enabling linear ubiquitin chains in the receptor signalosome [42].

Protease function is highly regulated *in vivo*; proteases can be translated as inactive zymogens that require removal of a pro-peptide for activation, and there is an extensive repertoire of endogenous inhibitors that restrict proteolysis. This makes the study of proteases by proteomics techniques challenging [43]. Mere quantification of proteins in a biological sample by shotgun-type approaches often lacks the information needed to infer conclusions on function, since it does not provide data on the activation status of the protease. After realizing this shortcoming, the field of degradomics sets out to develop more functional approaches to analysis of proteases in biological systems [44].

## 5. Applications of proteomics techniques to elucidate proteolytic events in lymphocyte antigen signalling

Since the function of a protease is defined not only by its activity status but particularly by the proteins it cleaves, several groups have dedicated their efforts to developing proteomics techniques for unbiased identification of protease substrates (reviewed in [45,46]). As with protease function itself, the occurrence of a cleaved substrate in a biological sample is often elusive to regular proteomics since the protein will still be identified through its tryptic peptides and additional information is needed to identify a protein as being cleaved. One strategy to overcome this was a natural consequence of a formerly popular proteomics method combining two-dimensional gel electrophoresis to separate proteins in a complex sample based on their isoelectric point and size, with MS identification of proteins after precise excision of individual protein spots. By comparing gels of samples incubated with the protease of interest to control samples, disappearance of certain spots, concurrent with appearance of lower-molecular-weight spots originating from the same protein, points to potential substrates. Although this technique has been used with some success in immunological research [47–50], the low reproducibility, labour intensity and lower coverage depth have largely made it redundant. A one-dimensional gel method named PROTOMAP was developed by the Cravatt lab to study apoptotic cleavages in Jurkat T cells [51,52]. One-dimensional electrophoresis pre-fractionates cell lysates after induction of apoptosis followed by LC-MS/MS identification of the proteins in each slice after in-gel digestion. By combining the MS identification and quantification by spectral counting with the molecular size information from the gel electrophoresis in a bioinformatics platform, they were able to map cleavages in 261 proteins in apoptotic cells, but this method is rarely able to pinpoint the exact cleavage site in the substrate.

Since bona fide substrates are uniquely identifiable by the novel or neo-N-terminal protein end of the cleavage fragment, methods that select for and enrich this peptide have been most successful (overview in figure 2*b*). Positive selection of the neo-N-terminal peptide after chemical labelling of the N-α-amino group by NHS-biotin seems like an attractive technique, but since normal chemical labelling conditions for α-amino groups will inadvertently also lead to modification of the lysine ϵ-amino group, specialized techniques are required to introduce selectivity. The Wells lab developed an elegant technique for selectively labelling α-amines with biotin by using a subtiligase enzyme to transfer a biotinylated peptide sequence containing a tobacco etch virus (TEV)-protease cleavage site selectively to the unmodified N-terminus of proteins [53]. After tryptic digestion, avidin–biotin enrichment of the labelled peptides and TEV-protease cleavage, the enriched N-terminal peptides are analysed by LC-MS/MS. Thus, 333 caspase cleavage sites in Jurkat T cells were identified in apoptosis, and their large database of N-terminal peptides and caspase cleavage sites is named Degrabase [54].

Owing to the technical difficulties in labelling protein N-termini selectively, the two currently most widely used N-terminomic techniques, combined fractional diagonal chromatography (COFRADIC [55,56]) and terminal amine isotopic labelling of substrates (TAILS [57,58]), rely on the opposite principle—negative selection. The Vandekerckhove and Gevaert labs developed COFRADIC by which all amines (α- and ϵ-) in a proteome are chemically acetylated prior to digestion by trypsin. Using the difference in acid dissociation constant of the acetylated (naturally or by the chemical labelling) N-terminal amino groups versus unmodified ones, COFRADIC negatively selects for N-terminal peptides by strong cation exchange (SCX) chromatography. Non-binding peptides are fractionated by reverse phase (RP) chromatography, and after a second labelling step of the internal tryptic peptides with free N-terminal α-amino groups in each collected fraction with 2,4,6-trinitrobenzenesulfonic acid (TNBS), RP fractionation negatively selects for the N-terminal peptides that have lower hydrophobicity compared with the TNBS modified internal peptides. The resulting fractions (approx. 100 per sample) are analysed by LC-MS/MS. By using isotopically labelled reagents for either labelling step, relative quantification between different samples is possible, and naturally and experimentally (i.e. neo-N-termini) acetylated peptides can be distinguished. COFRADIC provides advantages over the subtiligase approach; it allows for identification of naturally acetylated protein N-termini and it relies on only widely available chemicals and equipment. COFRADIC has been modified for improved yields and coverage [59–61] but remains dependent on many LC-MS runs per sample.

COFRADIC has been applied in several studies using lymphocytes to investigate substrates of members of the granzyme protease family. Granzymes are effector proteases used by cytotoxic T cells to neutralize pathogen-infected cells; upon recognition of MHC-presented antigen on an APC, cytotoxic T cells secrete granules containing granzymes and perforin that enter the APC and induce cell death. Lysates of SILAC labelled Jurkat T cells pretreated with pan-caspase inhibitors and then digested *in vitro* by granzyme-B, or cells exposed to natural killer cells, were analysed by COFRADIC [62]. More than 800 unique cleavage sites were identified in murine and human proteins. Generating a large cleavage dataset also allowed sequence specificity analysis, and the authors found interesting differences between the human and murine forms of granzyme-B, where the murine form has a strong preference for cleaving substrates before lysine residues. By sampling granzyme-B-incubated cell lysates at different time points, COFRADIC generated a basic overview of protease kinetics for different substrates [63]. Differential cleavage sequence preference between human and murine granzyme homologues was found for granzymes A, K and M [64,65]. COFRADIC-derived cleavage site specificity, combined with PROTOMAP analysis, found several granzyme-B substrates in basement membrane-associated protein, indicating that granzyme-B is also active extracellularly and involved in cytotoxic T-cell transmigration [66]. Granzymes are inducers of tumour cell death, and a COFRADIC study investigating granzyme-M in HeLa found 34 potential substrates. One of the substrates, topoll-α, was validated and cleavage was found to induce cell cycle arrest and apoptosis [67].

The most widely used N-terminal proteomics method based on negative selection of N-terminal peptides is TAILS, developed by the Overall lab. In TAILS, all α- and ϵ-amines are blocked at the protein level, by either reductive amination or amine-reactive NHS-based reagents. This step allows relative quantification between protease-treated and control samples by using isotopically labelled formaldehyde (e.g. ^13^CD_2_), or isobaric mass tags such as iTRAQ or TMT for different samples. The isotopically labelled samples are combined and digested by trypsin or another endopeptidase, e.g. GluC or lysarginase [68], which generates internal peptides with unblocked α-amines that are subsequently depleted by incubation with a commercially available approximately 100 kDa amine-reactive aldehyde-functionalized soluble hyperbranched polyglycerol polymer (HPG-ALD, http://flintbox.com/public/project/1948). Bound internal peptides are removed by filtration, and the unbound N-termini concentrated and analysed by LC-MS/MS. Unlike COFRADIC, TAILS does not depend on extensive fractionation and indeed performs better when analysed unfractionated, especially with the recent enhancements in MS speed and sensitivity. An aliquot of the sample prior to polymer depletion is used for a shotgun-like analysis on the same sample to normalize for differences in individual protein abundance between samples. TAILS has been successfully applied to studies on many proteases, especially members from the matrix metalloproteinase (MMP) family (reviewed in [69]). Originally thought to be primarily involved in degradation of extracellular matrix protein to enable cell migration and expansion, TAILS has revealed pleiotropic functions for many of these enzymes in immune-related processes by analysing inflamed skin and ankle joints, and inflammatory exudates in bronchioalveolar fluid and peritonitis from wild-type and knock-out mice [70,71], in antiviral responses [72] and the pancreatic RIP1-Tag cancer model [73]. TAILS has also been applied in haematopoietic cells such as platelets [74] and erythrocytes [75] to profile the N-terminome in these cells and to show that background proteolysis is far more pervasive as often thought, with up to 50–70% of the proteome having neo-N-termini.

N-terminal proteomics techniques are very powerful for targeted detection of substrate cleavage by endopeptidases and aminopeptidases, but certain proteolytic events are elusive. Sometimes this is due to a redundant neo-N-terminal sequence or otherwise an unfavourable peptide for MS analysis. Cleavage by carboxypeptidases and determination of the cleavage site after release of membrane-anchored proteins from the cell surface (so-called ‘shedding’) often mediated by the A disintegrin and metalloprotease enzymes (ADAMs, reviewed in [76]) require selective analysis of the C-terminal end of the protein. Several methods to achieve this have been published in recent years, such as a modified COFRADIC protocol [77] and a C-terminal specific version of TAILS (C-TAILS [78]), but results have not achieved the comprehensiveness of N-terminal proteomics. C-terminomics techniques are experimentally challenging since the carboxyl group is less reactive, and the methods rely on multiple additional labelling steps that increase losses and decrease sensitivity. A second phenomenon affecting the lower efficacy of C-terminomics is due to an inherent lower amenability of C-terminal peptides generated by trypsin to MS analysis since they lack a C-terminal basic residue. Use of alternative enzymes for digestion of the proteome prior to MS analysis can overcome this specific issue; cleavage by LysargiNase generates peptides with an N-terminal basic residue that have more favourable properties and improve the identification yield in C-TAILS [68].

## 6. Challenges in applying ubiquitin and protease proteomics to complex biology

Most PTMs only affect a fraction of the pool of the target proteins and are prone to variability between biological samples. Several recent technological advances in quantitative proteomics have aided in overcoming this issue and made probing the PTM landscape in complicated systems possible. Amine-reactive isobaric tags (TMT or iTRAQ) enable concurrent identification and multiplexed quantification of proteins in different samples. Increasing the number of isobaric tag-labelled samples that can be compared in a single experiment is highly desirable. However, it has been reported that increasing multiplexing by using larger tagging molecules, as in the case of iTRAQ4 versus iTRAQ8, decreases the number of identified and quantified proteins and peptides [79]. The development of neutron-encoded isotopologue variants of TMT has increased the multiplexing capacity to 10 samples [80] without increasing the size or altering the structure of the tag, and as a result, there is no loss of protein quantification from TMT6 plex to TMT10 plex [81]. These reagents use the 6 mDa mass difference between ^13^C and ^15^N isotopes, which can be resolved by modern high-resolution Fourier transform mass spectrometers. Recently, an approach was developed by the Coon lab for MS^1^-based quantification by metabolomics labelling that increases multiplexing capacity by using isotopologues of amino acids named neutron-encoded quantification (NeuCode [82]). The spacing between ‘light’ and ‘heavy’ isotopologues can be in the range of a few to a hundred mDa and requires MS^1^ mass resolving powers in excess of approximately 100 000. Since metabolic labelling has limited applicability in animal studies, a NeuCode carbamylation reagent was also developed [83].

Isobaric tag quantification by conventional tandem MS suffers from impaired quantitative accuracy and precision due to interference from contaminant peptides with similar chromatographic and mass-to-charge properties to the target peptide [84]. Improved quantitative performance can be achieved with an additional round of ion selection and fragmentation to purify the analyte from which isobaric tag quantification is derived [85], but such MS^3^-based methods result in substantially reduced sensitivity [86]. Synchronous precursor selection MS^3^ (SPS-MS^3^), a recently developed technique, which allows multiple peptide-specific fragments to be accumulated and increases reporter ion intensity to balance the quantitative gains of MS^3^ quantification with the sensitivity required for protein-wide analysis [87,88], has been applied to B-cell proteomic analysis [42]. An additional benefit of this approach is that, unlike conventional MS^3^, it is compatible with tryptic protein digestion; in the original MS^3^-based strategies from Ting *et al.* [85], LysC was used to guarantee that all MS^2^ y-ions contained a TMT tag [89]. There are several ways to improve sensitivity and selectivity of SPS-MS^3^ for PTMs. One option is to use different fragmentation techniques instead of collision-induced dissociation (CID), for example electron transfer dissociation (ETD [90]). ETD has the unique ability to access information about labile modification and provides extensive and complete fragmentation for larger and highly charged species, such as often observed in N-terminal proteomics, significantly benefiting identification of modified peptides [91].

Analysis of N-terminal proteomics data has proven to be challenging. To identify *in vivo* generated cleavages that will inherently yield peptides with a non- or semi-tryptic specificity, database searching has to be performed with more degrees of freedom, and the enrichment of peptides with N-terminal modifications such as acetylation or cyclization requires inclusion of these parameters as variable modifications, leading to both a larger search space and less reliable results. Several data analysis pipelines have been developed to improve robustness of identification, annotation and quantification of N-terminal proteomics data [92]. CLIPPER is an add-on to the Trans-Proteomics Pipeline (TPP [93]) that provides a tool for analysis of TAILS data combined with quantification by isobaric tags [94]. Knowledge of protease function is far from comprehensive; many human proteases have no known substrates. Annotation of protease function in databases such as MEROPS [95] and TopFIND [96,97] will increase our knowledge about the intricate interplay between proteases, inhibitors and substrates. With growing information density, these databases allow for functional predictions, and identification of specific protease activity from complex N-terminomics-derived datasets. Compared with the study of ubiquitination, proteomics investigation of proteases in adaptive immunity-related (patho-) biology has been lagging. One issue is that, although several proteases are involved in lymphocyte biology, most of these will be relatively promiscuous in nature, e.g. caspases, granzymes and proteases involved in MHC peptide generation and trimming. Although this will generate large degradomics datasets and provide insights into cleavage specificity, uncovering physiologically relevant key protease–substrate interactions is often challenging and requires careful mechanistic follow-up analyses. For example, after identification of a patient with a function-impairing mutation in the MALT1 gene [98], we used TAILS to investigate the proteolytic landscape in B cells derived from the patient compared with her healthy heterozygote sibling and mother in a 10-plex TMT study [42]. MALT1 is a highly selective protease, with only nine known substrates that are all involved in the regulation of NF-κB activation in B and T cells, and discovery of novel substrates by unbiased proteomics had proven intractable. Although the patient had severe immunodeficiency, the phenotype of the harvested B cells was not strong, so we applied the multiplexing capabilities of TMT10 to include sufficient experimental conditions. Two biological replicate experiments resulted in the clear identification of a new substrate validated in both B and T cells; HOIL1 cleavage led to disassembly of LUBAC with consequent decreased levels of linear ubiquitin conjugates in the cells. By analysing later time points than commonly performed for NF-κB activation studies, we showed for the first time that MALT1 was also a negative regulator of the BCR- and TCR-driven NF-κB response.

There were several interesting technical observations in this study. First, the difference in cleavage between the patient and one of the family member controls was insignificant when analysing the samples by MS^2^ quantification—only SPS-MS^3^ was able to visualize the partially abrogated cleavage in the patient cells. Secondly, the novel substrate HOIL1 was the only significant candidate substrate, whereas none of the known substrates were observed. This demonstrates the importance of choosing the biological experimental conditions wisely; N-terminomics methods only detect cleavage fragments that are still present, and many can be rapidly degraded after cleavage, such as known MALT1 substrate RelB. Using multiplexed labelling allows for correction of this by incorporating more time points in a single experiment.

## 7. Conclusion and outlook

In recent years, advances in both sample pretreatment techniques and enhanced LC-MS capabilities have opened up the field of targeted large-scale probing of PTMs by proteomics. What started with comprehensive profiling studies of phosphorylation has now been enabled for ubiquitination research, where techniques such as TUBEs and K-ϵ-GG antibody enrichment allow for identification of tens of thousands ubiquitination sites. This depth of coverage has enabled researchers to identify rare perturbations in ubiquitination events such as targets of very selective E3-conjugating enzymes or DUBs under specific (patho-) physiological conditions. Like ubiquitin research, proteomics of proteases has also matured by optimization of widely used techniques such as COFRADIC and TAILS. Our investigation in MALT1-deficient patient B cells by TMT10-TAILS has shown that with current technology it is possible to identify new key substrates of selective proteases and uncover new biological pathways. With the increasing availability of these powerful techniques, reagents and mass spectrometers, the possibility has arisen to probe multiple modifications in a comprehensive manner and truly paint a landscape of PTM in healthy and perturbed systems. Knowledge about pathways in adaptive immunity already show significant cross-talk between different PTMs, so these new technologies will undoubtedly lead to great new insights in the coming years.
